# Genome Array of Hair Follicle Genes in Lambskin with Different Patterns

**DOI:** 10.1371/journal.pone.0068840

**Published:** 2013-07-30

**Authors:** Wei Sun, Rong Ni, Jin Feng Yin, Hassan H. Musa, Tong jia Ding, Ling Chen

**Affiliations:** 1 College of Animal Science and Technology, Yangzhou University, Yangzhou, China; 2 Faculty of Medical Laboratory Sciences, University of Khartoum, Khartoum, Sudan; 3 Animal Science and Veterinary Medicine Bureau of Suzhou City, Suzhou, China; Northwestern University Feinberg School of Medicine, United States of America

## Abstract

Hu sheep lambskin comes from a specific breed of sheep of China. Hu sheep are considered a protected breed by the Chinese government. The hair follicles of these sheep have three types of waves, large, medium, and small. There are only few histological reports of Hu sheep lambskin, and there are no modern molecular or biological studies, so the molecular mechanisms underlying the formation of hair follicles with different patterns are not currently known. The aim of this article was to study the molecular mechanism of the formation of these types of hair follicles in Hu sheep. Histological and microscopic analysis indicated that the number of follicles with small waves was not significantly higher than the number of follicles with large waves (*P*>0.05). The diameters of primary and secondary small-wave follicles were significantly smaller than those of large-wave follicles (*P*<0.05; *P*<0.01). The ratio between the number primary follicles and the number of secondary follicles was significantly higher among small-wave follicles than among large-wave follicles (*P*<0.05). Differentially expressed genes in the skin tissue were screened using an Agilent gene chip and RT-PCR. Differential expression analysis revealed 3 groups of large waves and small waves; 1067, 2071, and 3879 differentially expressed genes; and 137 genes common to all 3 groups. Differentially expressed genes were classified using gene ontology. They were found to be mainly involved in cell differentiation, proliferation, apoptosis, growth, immune response, and ion transport. RT-PCR results of 4 differentially expressed genes were consistent with gene chip results. Combined with related literature, our results suggest that BMP7, MMP2, SNAI1, SFXN1, CDKNIC, MT3, and POU1F1 may have important effects on the formation of large-wave and small-wave hair follicles. This study may enrich knowledge of hair follicle development, and may identify the genes responsible for the formation of hair follicles with different patterns.

## Introduction

The hair follicle is a very complex structure whose behavior must be tightly regulated throughout its life. Histological structures of the follicles differ not only among different species but also across age groups and geographic area of origin within species [1]. Hair follicle induction and growth are dependent on interactions between the epidermis and underlying mesenchyme [2], [3]. Competition between various morphogenetic stimulatory (e.g., sonic hedgehog) and inhibitory proteins (e.g., bone morphogenetic proteins) eventually determines the final distribution and density of hair follicles [4], [5]. At birth, about 5 million hair follicles cover the surface of the body and no additional follicles form thereafter. The mature hair follicle, however, continues to grow in a cyclical fashion, progressing through 3 distinct phases, anagen (growth phase), catagen (regression phase), and telogen (resting phase) [6]. Hair cycling recapitulates follicular morphogenesis in many respects because it involves the proliferation and differentiation of epithelial stem cells in response to dermal papilla-derived signals [7], [8]. A number of additional anatomical, hormonal, nutritional, and genetic factors further modulate hair shaft size, shape, and color [9]. Several essential pathways, such as the Wnt, Shh, BMP, and FGF pathways, are essential to the reciprocal signaling events necessary for hair follicle morphogenesis and differentiation [10], [11].

Nagao et al. found that distinct parts of each hair follicle differed in their expression of ligands for CCR2 and CCR6. The isthmus expressed the chemokine CCL2; the infundibulum expressed the chemokine CCL20; and keratinocytes in the bulge produced the chemokine CCL8, which is the ligand for CCR8 [12]. Some genes that may play a role in hair follicle growth and development were also detected. These included the BMP gene [13], HOX gene, hr gene, and KAP gene [14], [15]. Kim et al. analyzed Hr^Hp^/Hr^Hp^ in the skin and built an expression map using gene chip technology to determine which genes were affected by excessive expression of HR. They found 8 genes to be associated with hair follicle development and confirmed the presence of RNA related to hair follicle formation in the skin by RT-PCR. Results showed that Wif1, Casp14, Krt71, and Sfrp1 genes maintained consistent expression patterns while all follicles overexpressed HR in vivo. HR may involve transcriptional regulation of these genes [16].

The use of DNA arrays for the monitoring of gene expression has become increasingly common in molecular biology. The ability of arrays to monitor thousands of separate but inter-related events simultaneously has intrigued scientists practicing both basic and applied research [17]. The single greatest scientific and technical challenge in creating a whole-genome microarray is to define which parts of the genome really correspond to genes or transcripts that will be represented on the whole-genome microarray [17]. The principal reasons for the increasing awareness of the molecular complexity of hair follicles are their position and visibility on the outside of the body, which makes phenotypic abnormalities readily apparent.

Hu sheep lambskin has three kinds of waves, large, medium, and small. The quality of small waves is excellent, and the quality of large waves is considered poor. Hu sheepskin enjoys an excellent reputation in the international fur market, where it is known as China's soft gem. The Hu sheep breed is protected by the Chinese government. However, in recent years, the economic value of Hu sheep has shifted from a focus on the skin as the main product with meat as the secondary product to meat as the main product with the skin as secondary. Because of this, the quality of Hu sheepskin has deteriorated considerably, and it has become almost impossible to find superior quality lambskin with the smooth pattern characterized by fluttering but not loose consistency. It is possible that genes responsible for this pattern may have been lost.

There are only few reports of histological research on Hu-sheep lambskin, and there are no modern molecular or biological studies, so no the molecular mechanisms of different patterns of hair follicle formation in Hu sheep are not currently known.

Identifying the genes responsible for the formation of large and small waves may allow the selective breeding of individuals capable of producing small-wave follicles. The aim of the present study was to investigate the differentially expressed genes of large waves and small waves using Agilent gene expression profiling technology and to screen genes involved in hair follicle cell differentiation, proliferation, apoptosis, growth, immune response, and ion transport.

## Materials and Methods

### Experimental populations

This study was carried out in strict accordance with the recommendations of the Guide for the Care and Use of Laboratory Animals of Jiangsu Province and of the Animal Care and Use Committee of the Chinese Ministry of Agriculture. The protocol was approved by the government of Jiangsu Province (Permit Number: 45) and the Ministry of Agriculture of China (Permit Number: 39). All efforts were made to minimize the animals' suffering.

Three pairs of full-sib individuals were selected at birth from among Hu sheep reared at a Suzhou stud farm in China. Each pair contained one individual with predominantly large-wave wool and one with predominantly small-wave wool. The waves were measured on the skin of the back. About 1.5 cm^2^ of surface wool was cut off and then cut into small pieces and placed into an RNA enzyme finger tube and stored in liquid nitrogen.

### Chip fabrication

The chip used for the test was a sheep whole-genome microarray provided by Shanghai Bohao Biotechnology Company. It contained 15,066 probes, representing approximately 12,716 genes.

### RNA extraction and purification

Total RNA was extracted in the presence of buffer containing β-mercaptoethanol and guanidine using an RNAiso plus kit in accordance with the manufacturer's instructions (Takara Biotechnology Dalian, Co. Ltd., China). RNA was eluted with 40 µl RNase-free water. Total RNA was measured using a Nano Drop ND-1000 Spectrophotometer (Nano Drop Technologies, Wilmington, Delaware, U.S.). Samples with purity (A260/A280) of >1.8 were used. Total RNA was purified using an RNeasy mini kit and RNase-Free DNase.

### Microarray hybridization

The purified RNA was labeled using an Agilent microarray expression profiling support kit, and the labeled RNA was purified using an RNeasy mini kit. According to the hybrid flow and supporting kit provided by Agilent microarray expression profiling facilities, the hybrid was rolled in a rolling hybridization oven at 65°C, and 10 rpm for 17 hours, and the volume of hybrid cRNA sample was 1.65 µg. The tablet was washed in a washing cylinder. The completed hybridization gene chip was removed from the hybridization oven, and the chip was washed with GE wash buffer and then scanned using an Agilent Microarray Scanner.

### Real-time PCR verification

Differentially expressed genes were selected randomly, and 18s served as a reference gene. The SYBR Green I method was used for quantitative testing to verify the reliability of the chip results. Relevant information about genes on RT-PCR and primers is shown in Table 1. Primers were designed by Oligo 7 using a chip probe corresponding to the nucleic acid sequence found here. cDNA was prepared using a PrimeSYBR® RT Reagent Kit and Perfect Real Time kit (Takara). The standard curve was established using cDNA gradient dilution, and each sample was tested 3 times in a 7500 PCR instrument for RT-PCR. The relative expression of the target gene was calculated according to the following formula: Relative expression = 2^−ΔΔCt^, ΔΔC t = (C, target gene –C, housekeeping gene)_large waves_– (C t, target gene–C, housekeeping gene)_small waves_.

**Table 1:** Relevant information of gene and primer sequences for RT-PCR.

Genes name	Primer sequences (5′→3′)	Produce size (bp)	GenBank accession No
18s	F:CCTGTAATTGGAATGAGTCCACTT	100	NM_001128154
	R:ACGCTATTGGAGCTGGAATTACC		
MMP2	F:GTACCCCAAGCCGCTGACC	116	NM_001166180
	R:TCCAGAATTTGTCTCCAGCGAAG		
MT-3	F:CTCCTGCACCTGCTCCGACTC	99	NM_001009755
	R:TCCTTGGCACATTTCTCGCACTC		
CTSF	F:AGGAGTTCCGTACCATCTACCTG	160	EE825413
	R:CAAGAGCCGCACATACCCTG		
BMP7	F:TGAGTTCCGCATTTACCAGG	177	DQ192015
	R:GTGGCTGTGATGTCAAAAAC		

### Data analysis

Feature Extraction Software 10.7 read date and Gene Spring Software 11.0 were used for data analysis. The read dates were normalized, and differentially expressed genes were screened with Ratio≥2 and Ratio≤0.5. To filter out unknown genes, functional annotations were searched using NCBI accession numbers. The SAS on-line system was used for cluster analysis, and the clustering results were displayed using the Tree View tool.

## Results

### Structural characteristics of hair follicle in Hu sheep

**Figure 1:**
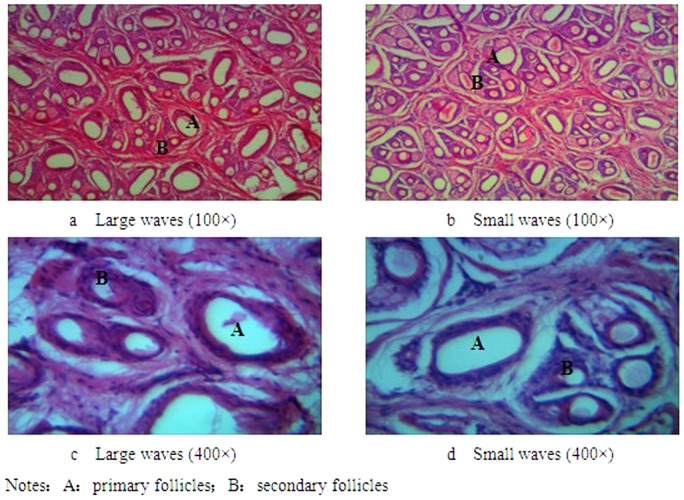
Cross section of skin tissue from the dorsal areas of Hu sheep.

The structural characteristics of hair follicles in the dorsal areas of Hu sheep were found to be clumped. Each group of hair follicles was found to be composed of 3 primary follicles and 1–2 secondary follicles. Most groups consisted of 3 follicles (Figure 1).

### Histological characteristics of large-wave and small-wave follicles

The ratio between the number of primary and secondary follicles, total number of follicles, and diameter of primary and secondary follicles is shown in (Table 2). Results indicated that the number of small-wave hair follicles was not significantly higher than the number of large-wave follicles (*P*>0.05). The diameters of primary and secondary small-wave follicles were found to be significantly smaller than those of large-wave follicles (*P*<0.05; *P*<0.01). The ratios between the numbers of primary and secondary follicles were significantly higher among small-wave follicles than among large-wave follicles (*P*<0.05).

**Table 2:** Ratios between the number of primary and secondary follicles, total number of follicles, and the diameters of primary and secondary follicles.

Group	Ratio between primary and secondary follicles number	Total follicle number	Diameter of primary follicles (µm)	Diameter of secondary follicles (µm)
Large waves	2.22±0.104a	59.67±1.484a	108.74±1.062a	49.85±0.420^A^
Small waves	3.24±0.217b	66.83±1.371a	98.24±1.023b	44.65±0.399^B^

Note: Means with different lower-case letters within the same column indicate significant differences between different rows. Means with different capital superscripts within the same column indicate extremely significant differences between different rows. Means with the same lower-case letter within the same column indicate no significant differences between different rows.

### Differential expression analysis

**Figure 2:**
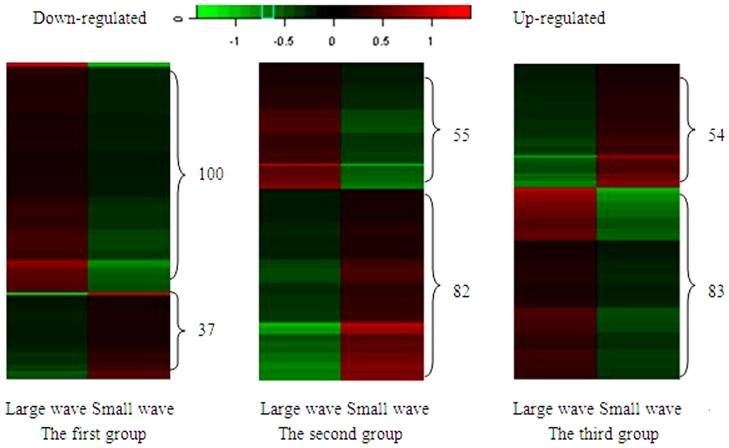
Cluster diagram showing approximately 137 differentially expressed genes across all 3 groups.

The experiment used full-sib matching design and the majority of Hu-sheep individuals are multiple births, we chose the differentially expressed genes by selecting common gene from the 3 pairs. The three pairs of sheep are here referred to as pairs 1, 2, and 3. In these pairs, differentially expressed genes were screened for differences greater than a factor of 2 or less than a factor of 0.5. After comparison, 137 genes were found to be common to all three groups. The arithmetic average about the individual differences in the expression levels of 3 pairs of genes in large-wave sheep and small-wave sheep was determined through logarithmic transformation with SAS online system, and a cluster chart was produced (Figure 2). In pair 1, 100 genes were up-regulated and 37 genes were down-regulated in small-wave follicles relative to large-wave follicles. In pair 2, 82 genes were up-regulated and 55 genes were down-regulated in small-wave follicles relative to large wave follicles. In pair 3, 54 genes were up-regulated and 83 genes were down-regulated in small-wave follicles relative to large-wave follicles.

Parts of differentially expressed genes are shown in Table 3. The expression of SFXN1, MT3, and MADCAM-1 was consistent in all three groups. The expression of other genes was only consistent in two groups.

**Table 3:** Partial sequences of genes differentially expressed between large-wave and small-wave follicles.

Gene name	GenBank accession No.	Fold change (FC) (first group/second group/third group	Gene ontology
UGT1A9	NM_001009189	8.4/3.46/0.1	Coumarin catabolism
SNAI1	DY499080	0.39/0.14/8.84	Cell apoptosis
SFXN1	NM_001126350	2.04/5.32/2.33	Red blood cell differentiation
PYGL	NM_001024861	2.43/0.29/0.27	Glycogen metabolism
POU1F1	NM_001009350	0.29/5.82/2.48	B cell differentiation
MT3	NM_001009755	0.43/0.29/0.29	Cell apoptosis
MMP2	NM_001166180	2.3/0.26/0.35	Cell adhesion
MADCAM-1	NM_001038020	0.46/0.13/0.22	Cell adhesion
LOC443512	DY490810	4.57/0.29/0.32	No comment
LOC443165	AF486295	0.5/0.4/0.08	No comment
LOC443080	EE752787	2.32/0.46/5.96	No comment
LOC100137070	EU366471	2.44/0.29/0.49	No comment
LOC100134932	U76740	3.23/0.22/4.46	No comment
H3F3A	EU366477	2.27/0.34/0.39	Nucleosome assembly
GLUT-1	U89029	2.54/0.08/2.12	Membrane transport
CYB561	NM_001093790	0.46/2.65/2.03	Transport
CXCL1	NM_001009358	2.42/0.41/2.02	Immune response
CTSF	EE825413	2.08/5.88/6.27	Protein hydrolysis
CSN2	NM_001009373	2.06/4.98/0.22	Transport
CITED2	EU340266	3.4/0.27/0.44	Bone morphogenetic
CDKN1C	NM_001142510	3.32/0.1/29.47	Glial cell differentiation
BMP7	DQ192015	2.64/0.19/3.33	Positive regulation of bone formation
ACTB	HM067830	0.34/0.42/10.57	Axon generation

Fold change (FC) ≥2 are up-regulated.

Fold change (FC) ≤0.5 are down-regulated.

### Gene ontology

**Figure 3:**
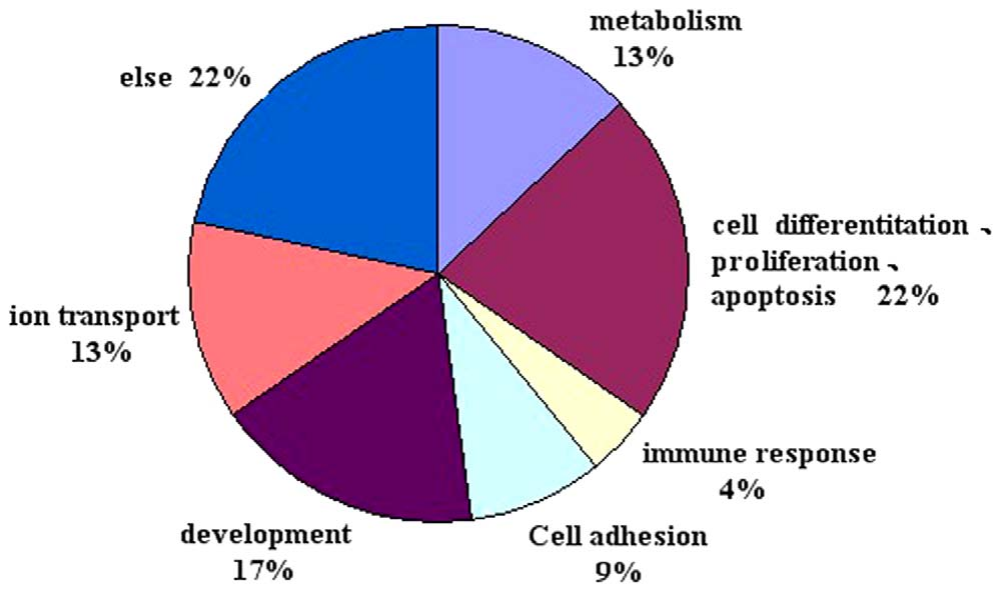
Classification of differential gene expression between large- and small-wave follicles.

The development of Hu sheep hair follicles is controlled by many genes, which are differentially expressed. Gene ontological analysis showed that these genes are mainly involved in cell differentiation, proliferation, apoptosis, growth, immune response, and ion transport. (http://www.geneontology.org/) (Figure 3).

### RT-PCR

**Figure 4:**
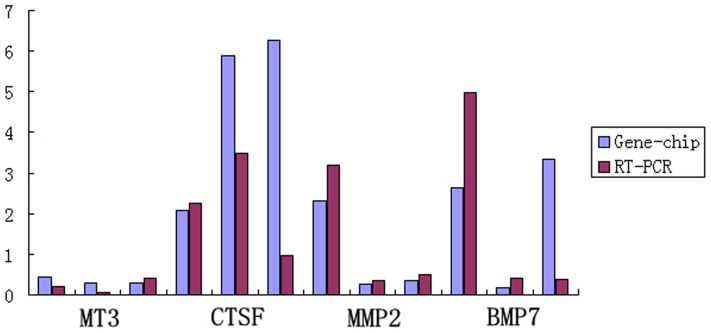
Microarray and RT-PCR.

In order to confirm the reliability of microarray results, 4 genes were selected for RT-PCR verification. The results show significant differences and some extremely significant differences between large- and small-wave follicles in pairs 1 and 2 (0.01<*P*<0.05) (*P*<0.01) (Table 4). Although microarray and RT-PCR indicated different fold changes, the expression trends were the same. This indicates that the microarray results are reliable (Figure 4).

**Table 4:** Microarray and RT-PCR results for the differentially expressed genes.

Gene	Groups	Microarray (fold change)	RT-PCR (fold change)	*P* value
MT3	1	0.43	0.20	<0.001**
	2	0.29	0.07	<0.001**
	3	0.29	0.41	0.046*
CTSF	1	2.08	2.26	0.018*
	2	5.88	3.49	0.017*
	3	6.27	0.964	0.668
MMP2	1	2.30	3.20	0.013*
	2	0.26	0.36	<0.001**
	3	0.35	0.50	0.003**
BMP7	1	2.64	4.97	0.023*
	2	0.19	0.41	0.01*
	3	0.33	0.39	<0.001**

Note:

* *P*<0.05, significance;

** *P*<0.01, extreme significance.

## Discussion

Hu sheep lambskin has several specific attributes, such as its wave pattern, white color, soft hair, clarity, and beauty. It contains three kinds of waves, large, medium, and small. The quality of small waves is considered far superior to that of large waves. In the present study, we used Agilent genome microarray technology to screen genes that showed differential expression in large-wave and small-wave follicles. In order to adjust for differences between individuals and environmental factors, we evaluated full-sibling individuals. The diameters of primary and secondary small-wave follicles were smaller than those of large-wave follicles, indicating that small-wave follicles offer high cashmere yield, less unmyelinated hairlessness from secondary follicles, and excellent general lambskin quality. Gene ontology was used to divide genes into three categories, cellular component, molecular function, and biological progress [18]–[20]. Branches in each category were found to contain different number of genes, and each gene could belong to different branches [21]. Differentially expressed genes observed in the present study showed that the expressed genes were mainly involved in development, cell differentiation, proliferation, apoptosis, immune response, and ion transport. These highly expressed genes may also be involved in hair growth and the hair follicle development.

Bone morphogenetic protein-7 (BMP7) is a member of the transforming growth factor-β (TGF-β) superfamily. The *in vivo* and *in vitro* experiments confirmed that TGF-β can stimulate hair follicle epithelial cells [22]. In developing and postnatal skin, BMPs, their receptors, and BMP antagonists show stringent spatio-temporal expression patterns. This facilitates proper regulation of cell proliferation and differentiation in the epidermis and in the hair follicle [23]. BMP2 is an induced inhibitor of hair follicles. It plays an important role in hair follicle formation and development. A related protein, BMP7 is also involved in cell differentiation. Expression of this protein is up-regulated in large-wave follicles, suggesting that BMP7 may be involved in hair follicle development or control the growth of its hair. Matrix metalloproteinase 2 (MMP2) is involved in the physiological processes of cell proliferation, differentiation, bone formation, and collagen catabolism, and it is a negative regulator of cell adhesion. It can shear and process insulin-like growth factor and then trigger the activity of insulin-like growth factor (IGF). It plays an important role in cell proliferation, differentiation, and inhibition of apoptosis [24]. SNAI1 is the inhibition of transcription factor snail superfamily member, may induce the occurrence of epithelial-mesenchymal transformation process, the studies show that SNAI1 can increase epithelial cell markers; high expression of this gene can resist hereditary cell death [25]. CDKNIC is a kinase inhibitor protein KIP family members, it can arrest cell proliferation in G0/G1 phase and has become a research hotspot in playing a negative regulation role in cell cycle in recent years. The present research on CDKNIC mainly focuses on cancer, the studies have shown that the specific inhibitor of miR-221 up-regulated CDKNIC expression, significantly inhibit the proliferation of tumor cells and induce the apoptosis [26]. MT3 is the encoding gene of metallothionein in cell, it has a unique molecular structure and high affinity with metal ion, not only has the physiological function of other MT, but also can inhibit cell proliferation [27]. POU1F1 is one of the POU domain factor families, it was thought to be a growth factor initially in the early time, it is a tissue-spcific transcription factor, and then it was confirmed to mainly regulate cell differentiation and the development of animal [28]. SFXN1 is a member of sideroflexin family and targeted to mitochondrial membrane. Some members of this family might be important in the carrier molecule and be related to the regeneration of pancreatic endocrine cells [29].These differentially expressed genes are all involved in cell proliferation, differentiation, and apoptosis. They may also be involved in follicle formation. Four differentially expressed genes were detected with RT-PCR and microarray and showed identical trends, suggested that gene chip technology is a reliable method of screening differentially expressed genes and that it can be used to provide strong evidence for subsequent experimentation.

### Conclusion

Approximately 137 genes were found to be differentially expressed among large- and small-wave follicles using gene chip technology. Gene ontology analysis indicated that some of these genes are involved in cell proliferation, differentiation, apoptosis, development, metabolization, and the immune response. The abnormal expression of BMP7, MMP2, SNAI1, CDKNIC, MT3, POU1F1 and SFXN1 showed that they may participate in the formation of hair follicles and so may produce the dorsal pattern. Some unknown genes were also detected. Their roles in hair follicle development have yet to be confirmed.
